# High concentrations of serum interleukin-6 and interleukin-8 in patients with bipolar disorder

**DOI:** 10.1097/MD.0000000000014419

**Published:** 2019-02-15

**Authors:** Yun-Rong Lu, Ying-Bo Rao, Yu-Jian Mou, Yan Chen, Han-Fen Lou, Yu Zhang, Dan-Xuan Zhang, Hai-Yan Xie, Li-Wei Hu, Ping Fang

**Affiliations:** a Department of Psychiatry, The Fourth Affiliated Hospital, Zhejiang University, School of Medicine, Yiwu; b Department of Psychiatry, The Second Affiliated Hospital, Medical School of Zhejiang University, Hangzhou; c Department of Clinical Laboratory, The Fourth Affiliated Hospital, Zhejiang University, School of Medicine, Yiwu, Zhejiang, China.

**Keywords:** bipolar disorder, C-reactive protein, interleukin 6, interleukin 8, major depressive disorder

## Abstract

Immune system dysregulation plays a key role in the physiopathology of bipolar disorder (BD) and major depressive disorder (MDD). However, whether interleukins might be biomarkers to distinguish these 2 affective disorders is unclear. Here, we assessed the differences in serum levels of interleukin 6 (IL-6) and interleukin 8 (IL-8) as well as C-reactive protein (CRP) in patients with MDD and BD. In total, we enrolled 21 MDD patients, 26 BD patients, and 20 healthy controls. We collected a total of 35 samples from BD patients in 3 different phases, depression phase, manic phase, and remission stage, and 27 samples from MDD patients in acute and remission phases. Serum IL-6 and IL-8 levels were assessed with solid phase sandwich ELISA-based quantitative arrays, and CRP levels were determined with an automatic analyzer. Both serum IL-6 and IL-8 levels were elevated in BD patients but not MDD patients. Subgroup analysis indicated elevated serum IL-6 in both the depression and manic phases in BD patients. The serum CRP levels did not change in either BD or MDD patients. However, sex differences in CRP concentrations were observed in healthy controls. Furthermore, there were linear correlations between the CRP levels and Bech-Rafaelsen Mania Rating Scale (BRMS) scores in BD patients. IL-6 and IL-8 levels may serve as biomarkers to differentiate between MDD and BD patients, even when the clinical manifestations are atypical. IL-6 may be used for the differential diagnosis of MDD and depressive episodes in BD.

## 1 Introduction

Mounting evidence suggests that aberrations in immune-inflammatory pathways contribute to the pathophysiology of mood disorders. Several mechanisms have been identified to explain the bidirectional relationship between mood disorders and immune dysfunction. On the 1 hand, activation of the immune system causes sickness behaviors that present during depressive episodes, such as low mood, anhedonia, anorexia, and weight loss,^[1]^ and neuroinflammatory illnesses are associated with emotional symptoms.^[2]^ On the other hand, substantial evidence has shown that major depressive disorder (MDD) and bipolar disorder (BD) are both related to peripheral inflammatory dysregulation.^[3,4]^

Peripheral inflammation markers are frequently reported in MDD^[5,6]^ and BD.^[7–9]^ Cytokines are cell signal transducing proteins or polypeptides that mediate and regulate immune responses and inflammation. Cytokines have been shown to cross the blood-brain barrier,^[10]^ thereby influencing many aspects of mood disorder pathophysiology, including neurotransmitter metabolism, neuroendocrine function, neural plasticity,^[11]^ and subsequently altering the activation of the brain and affecting emotion and behavior.

Interleukin 6 (IL-6) is an important proinflammatory cytokine. Some studies have found high levels of serum IL-6 in patients with depression,^[3,12]^ whereas other studies have reported no differences in IL-6 levels between patients with depression and controls.^[13,14]^ In addition, some studies have shown a significant decrease in IL-6 levels in MDD patients after antidepressant treatment.^[3,15]^ No change in IL-6, either in BD or in MDD, has been found in a recent study involving a Chinese population.^[4]^ Interleukin 8 (IL-8) is not only a proinflammatory cytokine but also a chemokine that can mediate the migration of inflammatory cells into inflammatory sites and effect the immune response in the acute inflammatory phase. Higher baseline proinflammatory cytokine (IL-6 and IL-8) levels and poorer antidepressant responses have been found in BD.^[16]^ However, other findings indicate that improvement in symptoms of depression and anxiety after treatment is not associated with changes in inflammatory markers.^[17]^ Elevated levels of the acute phase reactant C-reactive protein (CRP) are a very sensitive marker of acute inflammatory reactions. To date, CRP remains an interesting potential risk marker for BD, but its relevance remains to be established. Individuals with MDD may have elevated levels of CRP.^[18]^

The above results of studies on cytokines and CRP in patients with mood disorders are very inconsistent and contradictory. They are limited by heterogeneity between studies, insufficient standardization, and a lack of control for confounders in individual studies. Patients with depressive episodes of BD, as well as MDD subjects, may exhibit depressive symptoms, thus resulting in a high misdiagnosis rate.^[4]^ Further research exploring the roles of the peripheral cytokines IL-6, IL-8, and CRP in relation to different mood disorders is warranted. Furthermore, the heterogeneity of affective states and sex differences should be considered as an important confounder in studies. In addition, whether immune characteristics change after treatment remains unknown.

We hypothesized that unipolar depression and bipolar depression might present with different serum levels of cytokines (IL-6 and IL-8) and CRP even in the same affective state, and that mania and depression states might also present with different serum levels of cytokines and CRP. We sought to use these differences to identify different mood disorders and phases. Therefore, we performed a longitudinal study to identify differences in the serum levels of IL-6, IL-8, and CRP between MDD and BD in different phases before treatment, as well as any sex differences. In addition, we observed the trajectories of these cytokines and CRP in both types of patients during the acute attack and remission period.

## 2 Materials and methods

### 2.1 Study design

This was a single center, retrospective case-control study of patients with MDD or BD that took place between October 2014 and March 2018. This study was approved by the Institutional Ethics Committee of Fourth Affiliated Hospital, Zhejiang University School of Medicine, and was carried out in accordance with the Code of Ethics of the World Medical Association. All subjects provided signed written informed consent.

The primary aim of this study was to evaluate serum IL-6, IL-8, and CRP levels in both BD and MDD patients. Subgroup analyses were performed to explore the influences of different phases in the expression of serum IL-6, IL-8, and CRP proteins, and the value of these cytokines in distinguishing MDD from depressive episodes of BD. In addition, we also aimed to examine the sex differences in the expression of these proteins.

### 2.2 Participant population

In the present study, we reviewed data pertaining to patients diagnosed with MDD or BD at the Department of Psychiatry of the Fourth Affiliated Hospital, Zhejiang University School of Medicine (Yiwu, China) between October 2014 and March 2018. All patients were diagnosed with MDD or BD according to the Diagnostic and Statistical Manual of Mental Disorders, 5th edition (DSM-V) by qualified psychiatrists. The Mini International Neuropsychiatric Interview (MINI) (Chinese modified version)^[24]^ was used to confirm the DSM-V diagnosis. The inclusion criteria were

1) adult patients ≥18 years of age,
2) patients with first episode MDD or BD, and
3) patients not receiving any antipsychotics.

Healthy controls were screened for personal or family histories of neuropsychiatric disorders by using the Chinese version MINI^[24]^ and were included if they were free of these neuropsychiatric disorders.

The exclusion criteria were:

1) pregnant individuals,
2) individuals with serious suicide or impulse attempts,
3) individuals with current substance or alcohol abuse disorders,
4) individuals with previous attendance at or completion of one of the study treatments,
5) individuals with a primary diagnosis of schizophrenia or other psychiatric diagnoses involving Axis I and II disorders,
6) individuals with an abnormal body mass index (BMI ≤18 or BMI ≥25), and 7) individuals with any of the following comorbidities including acute and chronic infections, allergies, autoimmune diseases, cancer, and systemic diseases, antidepressant or antipsychotic treatment, immunomodulatory treatment, analgesic/anti-inflammatory use (e.g., acetylsalicylic acid, ibuprofen, or indomethacin), antibiotic therapy (e.g., hydralazine, tetracyclines, fluoroquinolones, quinolones, calcium, iron, chelating agents, or glucocorticosteroids), or substance abuse.

### 2.3 Clinical assessments

All subjects were evaluated through standard physical examinations, routine clinical laboratory tests, and psychological assessments when recruited. Clinical psychological assessments included the Hamilton Depression Scale (HAMD) (24-item),^[19]^ Montgomery-Asberg Depression Rating Scale (MADRS),^[20]^ Bech-Rafaelsen Mania Rating Scale (BRMS),^[21]^ Global Assessment Scale (GAS),^[22]^ and clinical global impression (CGI).^[23]^ The subjects were followed up once every 2 weeks or when their moods changed sharply. Psychological tests were assessed by professional psychological testing technicians. Depressive episodes were defined as HAMD ≥20 or MADRS ≥12, with BRMS <6. The remission stage was defined as HAMD <20, MADRS <12, and BRMS <6. Mania or mixed episodes were defined as BRMS ≥6. The investigation was conducted in accordance with the latest version of the Declaration of Helsinki.

### 2.4 Sample collection and measurements

After the first blood collection, all patients were treated with drugs according to clinical needs. During the follow-up, we collected the second and third blood samples when the patients entered another phase or remission. Fasting peripheral venous blood samples (5 mL) without anticoagulants were collected between 07:00 and 09:00 AM by venipuncture. The serum was obtained by centrifugation at 3000 rpm for 15 minutes. The serum was separated, divided into aliquots, and stored at −80°C in a refrigerator before laboratory assays. Serum levels of IL-6 and IL-8 were determined with ELISA kits (R&D Systems, Minneapolis, MN) in accordance with the manufacturer's instructions. Standard curves were constructed by using standard samples (IL-6 sensitivity of 0.7 pg/mL and IL-8 sensitivity of 7.5 pg/mL). We determined the optical density of each well within 30 minutes, by using a microplate reader (iMark Microplate Absorbance Reader, Bio-Rad) set to 450 nm, with wavelength correction set to 570 nm. Serum levels of CRP were measured with a Beckman Coulter AU680 automatic analyzer (Beckman Coulter). The CRP sensitivity was 0.2 mg/L. The assays were performed by the same technician, who was blinded to the sample's ID and clinical information.

### 2.5 Statistical analysis

Categorical variables are presented as frequencies. Continuous variables with normal distribution are expressed as mean ± SD. Continuous variables with non-normal distribution are expressed as interquartile range. Differences between groups are compared with chi-square test for categorical variables, with Student *t* test or analysis of variance with Bonferroni correction for continuous variables with normal distribution. The Mann–Whitney *U* rank sum test was used for continuous variables with non-normal distributions. Correlation analyses between continuous data were performed with Pearson correlation analysis. Spearman rank correlation analysis was used for correlation analysis between data with a non-normal distribution.

## 3 Results

### 3.1 Patient characteristics

In total, 21 untreated Chinese Han outpatients with MDD, 26 untreated Chinese Han outpatients with BD, and 20 age- and sex-matched control subjects were included in the present study. The MDD patients comprised 9 males and 12 females with a mean age of 39 years. The BD patients comprised 10 males and 16 females with a mean age of 34 years. The 20 age- and sex-matched control subjects comprised 8 males and 12 females with a mean age of 34 years. There were no significant differences in age and sex among the 3 groups. Most (>80%) of the participants were non-smokers (*P* = .43). The average durations of MDD, depressive episodes of BD, and mania/mixed episodes of BD showed no significant differences (*P* = .19). The onset time and the percentages of family history and psychotic symptoms were comparable among the 3 groups (*P* >.05). However, we observed that the suicide score was almost 3 times higher in depressive patients than mania/mixed patients (*P* = .034). The detailed information is presented in Table 1.

**Table 1:**
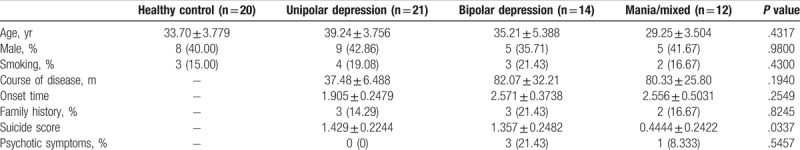
Patient baseline demographic and clinical characteristics.

### 3.2 Overall analysis of serum IL-6, IL-8, and CRP levels in patients with BD and MDD

**Figure 1:**
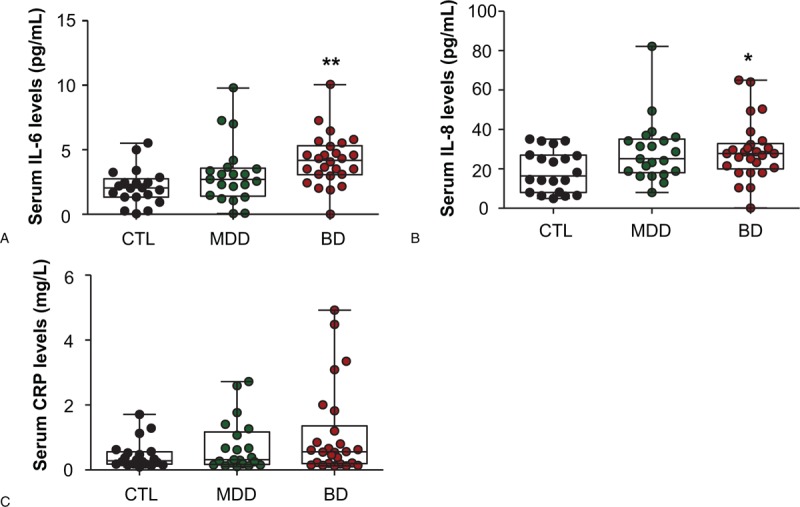
Serum levels of interleukin 6 (IL-6), interleukin 8 (IL-8), and C-reactive protein (CRP) in patients with bipolar disorder (BD) or major depressive disorder (MDD), and in healthy controls (CTL). The patients with BD had significantly higher IL-6 levels than CTL (A). The patients with BD had significantly higher IL-8 levels than CTL (B). No differences were found in CRP levels among the 3 groups (C). Data are shown as mean ± SD. ^∗^*P* <.05, ^∗∗^*P* <.01. CTL: healthy controls; MDD: major depressive disorders; BD: bipolar depression.

A significant difference in serum IL-6 levels were observed among the 3 groups of participants (*F* = 6.164, *P* = .0036; Fig. 1A). Further post-hoc analysis indicated that the serum IL-6 level was much higher in BD patients than healthy controls (4.191 ± 0.3879 vs 2.141 ± 0.3164, *P* <.01; Fig. 1A). Similar results were also observed for serum IL-8 levels, which were much higher in BD patients than healthy controls (29.10 ± 2.941 vs 18.96 ± 2.373, *P* <.05; Fig. 1B). However, there were no significant differences in serum CRP levels in BD patients (*P* >.05; Fig. 1C). In addition, no significant differences were observed in the cytokine serum IL-6 and IL-8, and in CRP in MDD patients (*P* >.05; Fig. 1A, B, and C).

In addition, we found linear correlations between CRP levels and BRMS scores in BD patients (ρ = 0.351, *P* = .039). However, no other relationships were found among IL-6, IL-8, and CRP levels and the clinical features of unipolar and bipolar depression (Table 2).

**Table 2:**
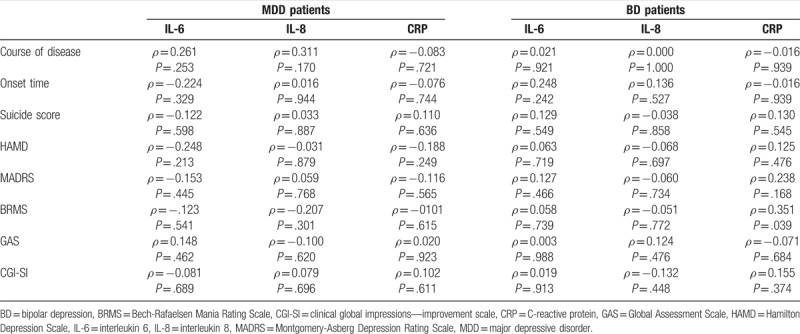
Correlation analysis between serum cytokines and clinical characteristics.

### 3.3 Subgroup analysis of serum IL-6, IL-8, and CRP levels in different episodes of BD and MDD patients

**Figure 2:**
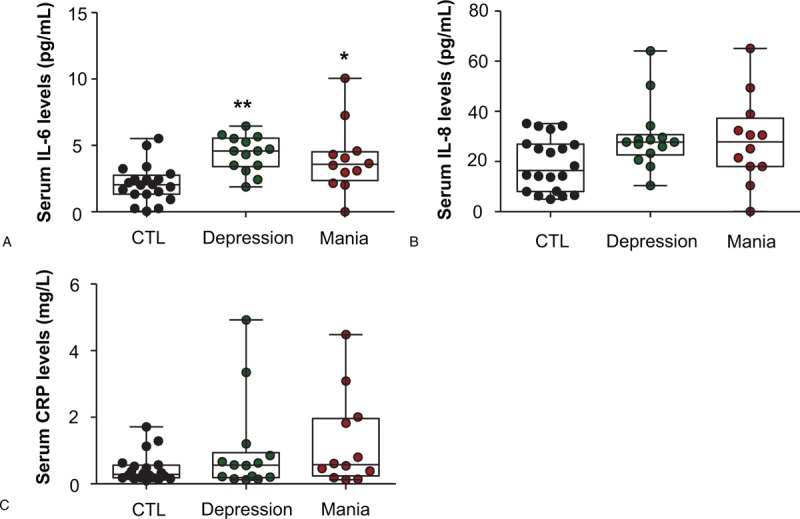
Serum levels of interleukin 6 (IL-6), interleukin 8 (IL-8), and C-reactive protein (CRP) in different phases in bipolar disorder (BD) patients. The patients in the depressive phase or mania/mixed phase of BD had significantly higher IL-6 than CTL (A). There were no significant differences in IL-8 (B) and CRP levels (C) among the 3 groups. Data are shown as mean ± SD. ^∗^*P* <.05, ^∗∗^*P* <.01. BDD, depressive episode of bipolar disorder; Mania, mania/mixed episode of bipolar disorder. Remission, remission period of bipolar disorder.

We sought to explore the value of these cytokines in distinguishing episodes of BD. We found that serum IL-6 levels showed significant elevation in both depressive (4.379 ± 0.3587 vs 2.141 ± 0.3164, *P* <.01; Fig. 2A) and manic/mixed (3.972 ± 0.7454 vs 2.141 ± 0.3164, *P* <.05; Fig. 2B) BD patients. There were no significant differences in serum IL-8 and CRP levels between depressive and manic/mixed BD patients (*P* >.05; Fig. 2B and C).

**Figure 3:**
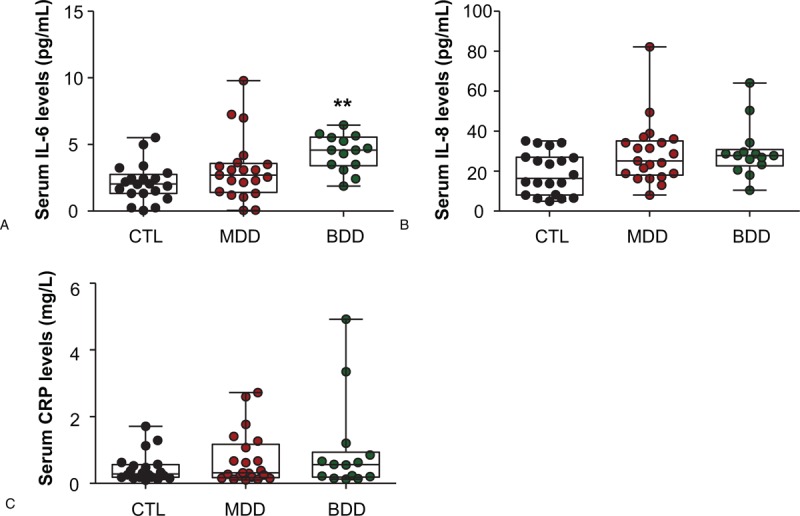
Serum interleukin 6 (IL-6), interleukin 8 (IL-8), and C-reactive protein (CRP) levels in patients with unipolar depression (MDD) or bipolar depression (BDD), and in healthy controls (CTL). IL-6 levels were elevated only in BDD (A) but not in MDD. There were no differences in serum IL-8 and CRP levels in the MDD, BDD, and CTL groups. (B and C). Data are shown as mean ± SD. ^∗∗^*P* <.01. CTL: healthy controls; MDD: major depressive disorders; BD: bipolar depression.

Furthermore, we examined the different expression levels of serum IL-6, IL-8, and CRP in BD and MDD patients. Interestingly, we found that serum IL-6 levels were higher in patients during depressive episodes of BD (4.379 ± 0.3587 vs 2.141 ± 0.3164, *P* <.01; Fig. 3A), but not MDD (3.105 ± 0.5200 vs 2.141 ± 0.3164, *P* >.05; Fig. 3A), than in healthy controls. However, no significant differences were observed in both serum IL-8 and CRP protein between MDD and depressive episodes of BD (*P* >.05; Fig. 3B and C).

### 3.4 Sex differences in expression of serum IL-6, IL-8, and CRP levels in BD and MDD patients

In the healthy controls, male participants showed higher serum CRP levels than female participants (0.5775 ± 0.2128 vs 0.3833 ± 0.8038, *P* = .024; Table 3). In both MDD patients and BD patients during depressive episodes, serum IL-8 was lower in female than male patients (*P* <.05; Table 3). In the manic/mixed BD patients, higher serum IL-6 levels were observed in male manic/mixed BD patients than female manic/mixed BD patients (3.784 ± 0.8232 vs 2.569 ± 0.3798, *P* = .0403; Table 3). In addition, we also observed higher CRP levels in male manic/mixed patients than female patients (2.116 ± 0.768 vs 2.569 ± 0.3798, *P* = .0403; Table 3).

**Table 3:**
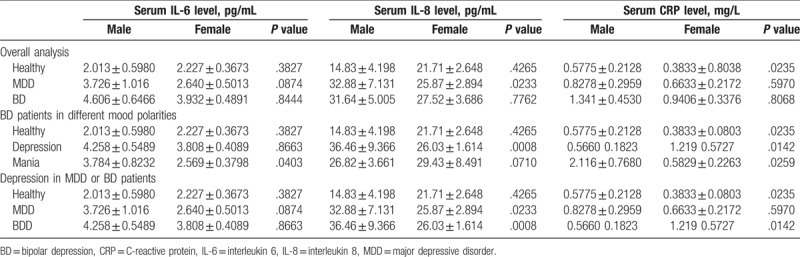
Gender differences in the expressions of serum IL-6, IL-8, and CRP proteins in MDD and BD patients.

### 3.5 Changes in IL-6, IL-8, and CRP levels after pharmaceutical treatment

**Figure 4:**
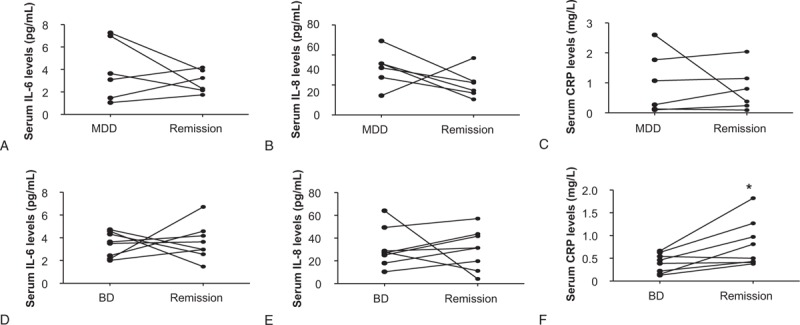
The changes in serum interleukin 6 (IL-6), interleukin 8 (IL-8), and CRP levels from an acute episode to the remission period in BD and MDD. The CRP levels in the remission period of BD were markedly higher than those in the acute attack phase (F). There were no significant changes in IL-6 and IL-8 levels from the acute episode period to the remission period of BD (D, E), and no significant changes in IL-6, IL-8, and CRP levels from the acute episode period to the remission period of MDD (Fig 4A, 4B, 4C). ^∗^*P* <.05. BD = bipolar disorder, CRP = C-reactive protein, MDD = major depressive disorder.

We further evaluated the changes in the serum levels of IL-6, IL-8, and CRP from acute episodes to the remission period in patients with MDD or BD. The CRP levels were markedly higher in the remission period in BD patients than in the acute attack phase (*P* <.05; Fig. 4F). There was no significant change in IL-6 or IL-8 levels from the acute episode period to the remission period of BD (*P* >.05; Fig. 4D and E); moreover, no significant changes were observed in IL-6, IL-8, and CRP levels from the acute episode period to the remission period of MDD (*P* >.05; Fig. 4A, B, and C).

## 4 Discussion

The present study indicated that the 2 kinds of mood disorders presented with different inflammatory features. Serum IL-6 and IL-8 levels were elevated in BD patients but not in MDD patients. Furthermore, serum IL-6 levels were high in both depressive and manic/mixed BD patients and thus may help to distinguish the diagnosis of MDD and depressive episodes of BD. However, no significant correlations were observed between serum IL-6 and patient characteristics including disease course, onset time, suicide score, and scale scores. In addition, sex differences were also found in the present study; we observed high levels of serum CRP in healthy male controls, serum IL-6 in male manic/mixed BD patients, and serum IL-8 in both male MDD patients and BD patients during depressive episodes.

First, the present study showed that serum IL-6 levels were elevated in BD patients, whether in the depressive phase or mania/mixed phase, but not in MDD. Evidence exists for an association between IL-6 and depressive episodes, both in bipolar and unipolar depression.^[3,18,25–28]^ Our study reported that serum IL-6 levels increased not only in depressive episodes but also in mania or mixed episodes. This is the first report of sex differences in IL-6 in BD patients. We believe that serum IL-6 might be a potential marker for the differential diagnosis of BD and MDD, especially for distinguishing MDD and depressive episodes of BD.

Second, we found that patients with BD showed higher levels of IL-8 than healthy controls. IL-8 may be more closely related to BD than MDD. Previous studies on the variation of IL-8 in mood disorders have been inconsistent, possibly because of the large variations in IL-8 concentrations in plasma.^[14]^ In agreement with our results, 1 study has found no significant differences in IL-8 between MDD patients and healthy individuals.^[14]^ Another study has found lower levels of IL-8 in patients with MDD.^[29]^ Several studies have suggested that imbalanced IL-8 levels, whether elevated or reduced, may be due to whether the patient has obvious anxiety symptoms.^[29,30]^

Third, we compared the changes in serum IL-6, IL-8, and CRP before and after treatment, and found no significant changes, except for a further increase in CRP in BD patients in remission phase compared with the acute attack period. Our study also found that the serum CRP levels were elevated only in the male mania/mixed group of BD patients. Furthermore, there was a linear correlation between CRP levels and BRMS scores in patients with BD. Owing to the relatively small sample size; we did not divide depression episodes and manic episodes into subgroups when we analyzed the changes in acute and remission periods. This design may be the main cause of the paradoxical results. However, we suggest that CRP is closely related to manic episodes, especially in male patients. CRP, a general laboratory marker of immune activation and inflammation, was used as a non-specific inflammatory biomarker. Age, smoking, and BMI was considered as covariates.^[31]^ Previous meta-analysis reports have indicated that CRP levels are significantly higher in manic and euthymic patients, but not depressed patients with BD than in controls.^[32]^ This finding is in line with our results.

No significant correlation of inflammatory factors with suicide symptoms was observed, a finding inconsistent with previous studies indicating that IL-8 levels are low in patients with depression and anxiety who attempt suicide.^[33]^ This finding is probably due to the exclusion of particularly serious suicide cases in our study. No significant correlations of IL-6, IL-8, or CRP with the course of disease or onset times were observed, a result inconsistent with previous studies indicating that a long duration of the disorder may lead to the release of chemokines;^[34]^ this inconsistency may be a result of our short follow-up period.

In the clinic, it is difficult to discern the emotional state of some patients, who might feel unhappy but have more speech, complaints, temper tantrums, and insomnia, or who may be excited but simultaneously have a frequent desire and impulse to commit suicide. These patients are easily misdiagnosed, and consequently, inappropriate treatment leads to a faster transition to serious mania or depression. Currently, although biological criteria are not used for the diagnosis of mood disorders, the finding that different inflammatory patterns exist in MDD and BD may provide a hint for further investigations exploring candidate biomarkers for distinguishing these 2 disorders. Our findings should help to differentiate these atypical patients clinically; for example, elevated IL-6 may distinguish between BD and MDD.

There were some limitations in this research. First, the sample size was relatively small. Second, owing to the small sample size, we did not further study subgroups based on different drugs. Third, for ethical reasons, we did not recruit patients with a serious urge to commit suicide or serious aggression. In addition, although we controlled for some confounders, peripheral inflammatory cytokines may also be affected by other factors, such as stress^[35]^ and physical activity.^[36]^

In summary, we investigated the serum levels of IL-6, IL-8, and CRP in Chinese MDD and BD patients during different mood states. We found that these patients presented with different immune features in the same affective state, and IL-6 may be used for the differential diagnosis of BD and MDD, and may be especially useful in distinguishing mania/mixed and depression even when the clinical manifestations are atypical. In addition, the relationship observed between CRP and remission state may provide some clues for the development of new therapeutic drugs and individualized strategies in the future. Because our results might partially be attributable to the relatively small sample size, further studies with larger sample sizes should be conducted in China in the future.

## Acknowledgments

We would like to thank all patients who participated in our study. Special thanks to Dr Li-Gen Shi, Dr Yi-Xiong Zheng, and Dr Er-Qing Wei for their supervision in writing and data analysis.

## Author contributions

Yun-rong Lu conceived and designed the experiments. Ying-bo Rao and Yu Zhang performed the sample collection, solid phase sandwich ELISA-based quantitative arrays and CRP analysis. Yu-Jian Mou and Han-Fen Lou conducted the psychological tests. Hai-Yan Xie, Yan Chen, Dan-Xuan Zhang and Ping Fang were responsible for the patient recruitment and follow- up. Li-Wei Hu contributed to the data analysis, and Yun-Rong Lu wrote the first draft of the manuscript. All authors contributed to and have approved the final manuscript.

**Data curation:** Ying-Bo Rao, Han-Fen Lou, Ping Fang.

**Formal analysis:** Han-Fen Lou, Hai-Yan Xie, Ping Fang.

**Investigation:** Yu Zhang.

**Methodology:** Yu-Jian Mou, Yan Chen, Yu Zhang, Ping Fang.

**Project administration:** Yunrong Lu.

**Software:** Yan Chen, Dan-Xuan Zhang.

**Visualization:** Dan-Xuan Zhang, Hai-Yan Xie.

**Writing – original draft:** Li-Wei Hu.
